# Sports-Related Injuries in Deaf Competitive Squad Athletes—Results of a Retrospective Self-Assessment

**DOI:** 10.3390/sports13020043

**Published:** 2025-02-06

**Authors:** Bastian Mester, Kim Lennartz, Julia Kristin, Heinz-Lothar Meyer, Christina Polan, Monika Herten, Marcel Dudda, Manuel Burggraf

**Affiliations:** 1 Department of Trauma, Hand and Reconstructive Surgery, University Hospital Essen, Hufelandstraße 55, 45147 Essen, Germany; kim.lennartz@stud.uni-due.de (K.L.); heinz-lothar.meyer@uk-essen.de (H.-L.M.); christina.polan@uk-essen.de (C.P.); monika.herten@uk-essen.de (M.H.); marcel.dudda@uk-essen.de (M.D.); 2 Department of Otorhinolaryngology, University Hospital Düsseldorf, Moorenstraße 5, 40225 Düsseldorf, Germany; julia.kristin@med.uni-duesseldorf.de; 3 Department of Orthopedics and Trauma Surgery, BG-Klinikum Duisburg, University of Duisburg-Essen, Großenbaumer Allee 250, 47249 Duisburg, Germany; 4 Department of Orthopedics and Trauma Surgery, GFO Kliniken Mettmann-Süd, Klosterstraße 32, 40764 Langenfeld, Germany; manuel.burggraf@gfo-kliniken-mettmann-sued.de

**Keywords:** Deaflympics, deaf sports, downtime, hard of hearing, injury rate

## Abstract

Background: Deaf squad athletes regularly participate in national/international competitions and most prepare for competitions in clubs with hearing athletes. Hearing loss is associated with difficulties in balance control which may impair functionality. The aim of this study is to provide epidemiological data on sports injuries in deaf squad athletes. Methods: In this retrospective study, data on main sport, training and competitions, injury rates, and downtimes were collected. Questionnaires from *n* = 65 athletes (*n* = 15 females, *n* = 50 males; age 28.00 ± 0.40 years) were finally analyzed. All injuries during the athlete’s career within nine body regions were recorded. The influence of contact sports, supervision by Olympic Training Center (OSP), and hearing aids on injury rates and downtimes were analyzed. Results: An amount of 89.20% were additionally registered in a regular sports club. A total of 1430 injuries were recorded during 465,400 training hours (3.07 injuries/1000 training hours). The highest prevalences were found for hand/fingers (43.00%) and ankle/foot (14.00%). Contact sport athletes had higher injury rates and longer downtimes (5.66 versus 1.28 injuries/1000; *p* < 0.001; 2.45 versus 1.11 weeks/1000; *p* = 0.011). OSP athletes showed lower injury rates (0.92 versus 4.38 injuries/1000, *p* = 0.004). Longer downtimes were recorded for athletes without hearing aids during training (2.29 weeks/1000 versus 0.96 weeks/1000; *p* = 0.045). Conclusions: Deaf athletes are exposed to additional training and competition in hearing sports. The negative impact of contact sports may be attributed to hearing loss. Hearing aids in training seem to be protective. Athletes should consider supervision by OSP. The results of this study may help to improve treatment and integration of deaf athletes into hearing sports.

## 1. Introduction

People with deafness or profound hearing loss claim to have a distinct cultural identity and belong to a sociolinguistic community, but the majority do not see this as a disability [1,2,3]. Although the hearing society generally perceives deaf individuals as disabled, culturally Deaf individuals view their deafness with pride because it indicates common social customs, language, and identity [4,5,6]. Contrarily, being deaf or hard of hearing is often considered a positive attribute or gain rather than a disability [3,7].

Most deaf and hard of hearing people who participate in recreational sports prefer to compete against their peers rather than against those who can hear [8,9]. In contrast to this, Kurkova et al. revealed in semi-structured interviews with European elite deaf athletes that 77.40% participated in competitions with hearing athletes, and 50.00% did not prefer deaf versus hearing competitions [9]. Most of the participants stated that they prepare for competitions in regular clubs with hearing athletes as well as in clubs for deaf athletes, resulting in a double exposure.

Nationwide, the German Deaf Sports Association (“Deutscher Gehörlosen-Sportverband”, DGSV) supports about 170 squad athletes who participate in national and international competitions and, depending on their performance, in an event equivalent to the Olympic Games—the Deaflympics—[10]. In 2025, the 25th Summer Deaflympics will take part in Tokyo, Japan. The International Committee of Sports for the Deaf (ICSD) serves as the international body that plans and organizes the competitions of the Deaflympics [1].

The DGSV squad athletes fulfil the criteria for deafness according to the guidelines of the ICSD. Participants in the competitions must be deaf defined as a hearing loss of at least 55 dB Pure Tone Average (PTA) in the better hearing ear, which has to be verified by an audiogram [11]. Athletes therefore fall into the categories of “severe hearing loss”, “profound hearing loss”, and “complete hearing loss” as defined by the World Health Organization (WHO) [12].

Although some research has been carried out on biomechanical parameters, functional movement analysis, and cardiorespiratory performance of deaf athletes in comparison to hearing athletes [13,14,15,16,17,18,19,20,21,22], there is a lack of literature on the epidemiology of injuries and overuse syndromes in athletes with a hearing loss. This fact may be explained by the heterogeneity of the hearing loss, and that people with a hearing loss mostly have a communicative challenge [1].

On the other hand, evidence regarding sports-related injuries in athletes with disabilities in general is also limited. In 2014, Fagher et al. published a systematic review on sports-related injuries in athletes with disabilities [23], including 25 studies dealing with this issue. Few studies were disability-specific, making it difficult to determine specific risk factors, and few studies reported detailed injury type and severity. The authors conclude that disability-specific studies are needed to identify and prevent injuries in athletes with disabilities.

Ferrara et al. examined injuries in athletes with disabilities in a review of the literature, analyzing injury data from Paralympic competitions dating back to 1976 [24]. One main finding was that injury patterns among athletes with disabilities appear to be like those of athletes without disabilities. Abrasions, strains, and contusions were more common than fractures and dislocations, as expectable. However, the affected body region appeared to be disability- and sport-dependent. The authors criticize a certain data collection bias and lack of homogeneity within the available studies and request future projects to present a realistic description of sport injuries.

Hearing loss has been shown to be associated with difficulties in balance and postural control [17,25,26,27,28]. In the context of road traffic injuries among deaf or hard of hearing people, it is known that this population group has difficulties in localizing sounds and in separating sounds from motor vehicles and buses. This can lead to a higher risk of experiencing pedestrian injuries [29,30,31].

Extrapolating these data to sports and recreation, these limitations may negatively affect motor control and impair functionality in sports [32,33,34,35]. Recently, Zarei et al. published a systematic review and meta-analysis on balance control in deaf or hard of hearing individuals, including 24 studies and 27 trials with a total of 2148 participants [36]. It was shown that in the physically highly active age groups between four- and 22-years deaf individuals had significant balance deficits compared to hearing controls. The meta-analysis revealed that hearing individuals had better balance than deaf individuals who were active in sports. Consequently, a relevant number of injuries and downtimes may occur in deaf recreational as well as competitive sports, which could be attributed to sports activity as well as deafness itself.

The aim of this unique study is to provide epidemiological data on injuries and sport-specific conditions in deaf athletes. Up to now, this issue has not been scientifically analyzed; similar research is not available. We hypothesize that deaf squad athletes of different sports disciplines suffer a relevant number of injuries, resulting in significant downtimes. Additionally, we hypothesize a correlation of sports-related variables (contact sports, supervision by an Olympic Training Center) as well as deafness-related variables (use of hearing aids) on injury rates, downtimes, incapacities for work, and medical consultations.

## 2. Materials and Methods

In this retrospective cross-sectional study in cooperation with the DGSV, injuries and sport-specific conditions in deaf sports were investigated.

A self-developed online-based questionnaire (Limesurvey version 6.10.0+250106; LimeSurvey GmbH, Hamburg, Germany) was developed, which has already been used in modified versions by our study group in the past [37,38,39,40,41] and has shown its clinical applicability. Similar research has been conducted before, regarding sports-related injuries in athletes with disabilities [23], and the recent study protocol was adjusted to established research tools also conducting the study by an online-based questionnaire [42]. Data on demographics, main sport, training behavior, and participation in competitions were collected from deaf squad athletes. In addition, the number and type of injuries and associated downtimes were queried.

The study was approved by the local Ethics Committee of the University of Duisburg-Essen (No. 20-9761-BO, 17 May 2021).

### 2.1. Study Population

A total of 170 squad athletes from different sports disciplines who are supervised by the DGSV were contacted and invited to participate in the study. The inclusion criterion for participation in the study was sports participation as a deaf person (internationally valid definition according to ICSD and WHO [11,12]) with organization at club level as well as support as a squad athlete by the DGSV.

Finally, sufficiently completed questionnaires from *n* = 65 deaf squad athletes (*n* = 15 females, *n* = 50 males; mean age 28.00 ± 10.40 years) were included in the data analysis, resulting in a response rate of 38.20%. All subjects were informed about the study and agreed to participate by signing an informed consent form in accordance with the tenets of the Declaration of Helsinki and the ethical standards in sport and exercise science research [43].

**Figure 1:**
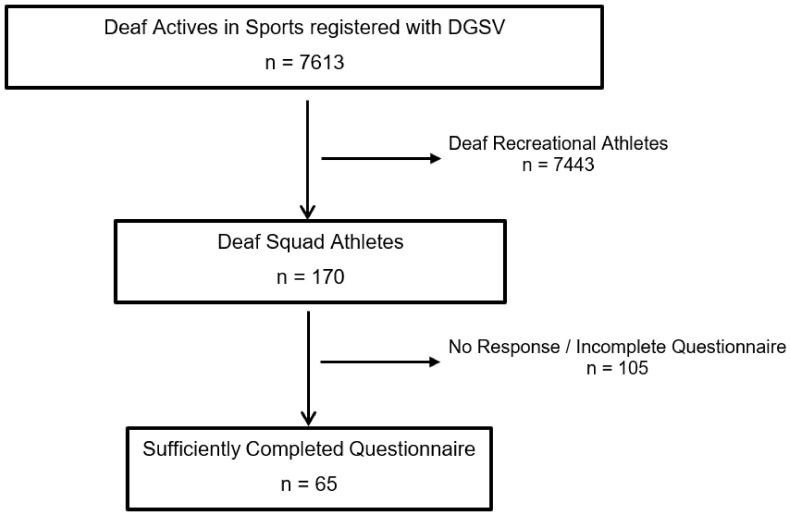
CONSORT diagram presenting the enrolment of the final study population according to [44]. Inclusion and data analysis of *n* = 65 deaf squad athletes.

Figure 1 shows the subject enrolment process and the application of inclusion and exclusion criteria.

### 2.2. Online-Based Questionnaire

The questionnaire consisted of 16 pages with a total of 127 questions and was divided into a general and a specific section.

The general section of the survey was designed to provide a comprehensive overview of the study population and to identify potential risk groups for subsequent statistical analysis. It included information on basic demographics, main sports discipline and level of competition, squad affiliation, institutional support by an Olympic Training Center (“Olympiastützpunkt”, OSP) of the German Olympic Sports Confederation (“Deutscher Olympischer Sportbund”, DOSB), training behavior, participation in training and/or competitions in hearing sports, and data regarding timing of injury (training versus competition, deaf versus hearing sports) and resulting downtime in sports, incapacity for work/school, as well as medical consultations in an average year.

The specific section of the questionnaire asked about all injuries and overuse syndromes sustained during the deaf athlete’s athletic career. All soft tissue, bone, and ligament injuries sustained during training and competition were included. The occurrence of a total of 58 injuries and overuse syndromes was specified in nine anatomical regions: head, spine, trunk, shoulder, elbow, hand/fingers, hip/thigh, knee, ankle/foot. These body regions were chosen concordantly to previous research on sports injuries and overuse syndromes conducted by other authors and our own study group [37,38,39,40,41,45,46] and therefore can be regarded as a commonly used standard.

Specifically, the following injury entities were recorded as totals for the nine body regions:

- Head: Abrasion/laceration, concussion, intracranial hemorrhage, nasal fracture, skull fracture;
- Spine: Lumbago, herniated disc, vertebral body fracture;
- Trunk: Abdominal muscle strain, rip contusion, rib fracture;
- Shoulder: Shoulder dislocation, contusion, adhesive capsulitis, biceps tendinopathy, impingement syndrome, rotator cuff tear, proximal humerus fracture, clavicle fracture;
- Elbow: Contusion, elbow fracture, elbow dislocation, tendinitis/epicondylitis;
- Hand/fingers: Cut/blister/abrasion, contusion, wrist fracture, hand fracture, tendon injury, tenovaginitis, finger sprain, finger fracture, finger dislocation, carpal tunnel syndrome;
- Hip/thigh: Pelvic contusion, pelvic fracture, femoroacetabular impingement, acetabular labral injury, femur fracture, thigh contusion, muscle strain;
- Knee: Contusion, distortion, cruciate ligament tear, collateral ligament injury, meniscal tear, cartilage damage, patella dislocation, patella tendinopathy, other tendinopathies;
- Ankle/foot: Contusion, fracture of the lower thigh, muscle strain, Achilles tendon rupture, achillodynia, ankle fracture, ankle and foot ligament rupture, foot fracture, toe fracture.

Consistent with the general section, data on the timing of injury and resulting downtime, incapacities for work/school, as well as medical consultations, were evaluated separately for each anatomical region.

The online-based questionnaire as raw version is available as Supplementary Material (Online-Questionnaire_German).

### 2.3. Statistical Analysis

Statistical analysis was carried out using Microsoft^®^ Excel^®^ for Microsoft 365 version 2412 (Redmond, WA, USA) and IBM^®^ SPSS Statistics software version 28.0.0.0 (IBM, Armonk, NY, USA). Sample size calculation was not performed, as all available squad athletes supervised by the DGSV were invited to participate in the study, and the total sample size is limited by the definition of this very selective population. All values were tested for normal distribution using the Kolmogorov–Smirnov test. Descriptive statistics included mean value ± standard deviation for normally distributed data and median [first quartile; third quartile] for non-normally distributed data, respectively. Data were analyzed by calculating percentages and injury rates per 1000 training hours. An extrapolation on 1000 h of exposure can be regarded as a commonly used standard in scientific analyses of sports injury rates and was chosen to enhance comparability with the available literature [47,48,49,50,51]. In addition to the descriptive evaluation, the influence of the variables contact sports, supervision by an OSP, and use of hearing aids on injury rates, downtimes, incapacities for work/school, and medical consultations was investigated with regard to group differences. As all data regarding the testing for group differences were non-normally distributed, the Mann–Whitney-U test was utilized.

Effect size intervals for the tests and respective effect size results were calculated by Pearson correlation coefficient (r). Interpretation of effect size was carried out according to the distribution as follows: r = 0.10 indicates a small effect; r = 0.30 indicates a medium effect; r = 0.50 indicates a large effect [52].

For all inferential statistical tests, the significance level was set at *p* < 0.05 and the level for highly significant results was set at *p* < 0.001.

## 3. Results

The study population comprised 65 deaf squad athletes (15 females, 50 males) with a mean age of 28 ± 10.40 (min 13.00, max 62.00) years. The subjects were of normal weight with a mean Body Mass Index (BMI) of 23.43 ± 3.06 kg/m^2^ according to the global WHO recommendation of 18.50–24.90 as a normal BMI [53].

**Figure 2:**
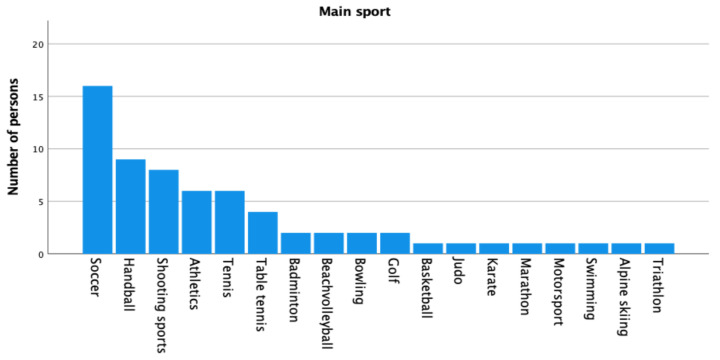
Distribution of the stated main sport in the collective of deaf squad athletes. Indication of 18 different disciplines, most frequently Soccer *n* = 16, Handball *n* = 9, Shooting sports *n* = 8. Athletics *n* = 6, Tennis *n* = 6, Table tennis *n* = 4, Badminton, Beach volleyball, Bowling, Golf *n* = 2 each, Basketball, Judo, Karate, Marathon, Motorsport, Swimming, Alpine skiing, Triathlon *n* = 1 each.

With a total of 18 different disciplines, soccer was the most frequently practiced sport in the collective (24.60%), followed by handball (13.80%) and shooting sports (12.30%) (see Figure 2).

The athletes had been practicing their main sport discipline for a median of 14.00 [first quartile 10.00; third quartile 20.00] years. An amount of 67.70% (*n* = 44) of the athletes had participated at least once in international competitions (European Championships, World Championships, Deaflympics).

An amount of 89.20% (*n* = 58) were also registered in a regular sports club in addition to deaf sports, 98.30% of which also take part in competitions with hearing athletes. These “double exposed” athletes had a higher median total training time per week of 8.50 [5.00; 14.25] hours, versus 5.00 [2.00; 7.00] hours in deaf sports only. Although a statistical trend was demonstrated, this difference was not significant (*p* = 0.066). The median weekly training frequency of athletes exclusively active in deaf sports was 2.00 [2.00; 3.00] sessions. In contrast, dual-sports athletes had a median of 1.00 [1.00; 2.00] training sessions per week in deaf sports and an additional 3.00 [2.00; 5.00] training sessions per week in hearing sports.

Overall, significantly more competitions per year are played in hearing sports than in deaf sports (16.00 [8.00; 30.00] in hearing sports versus 4.00 [2.00; 10.00] in deaf sports; *p* < 0.001).

### 3.1. Total Injuries and Injury Locations

A total of 1430 injuries were recorded during 465,400 training hours, which corresponds to 3.07 injuries per 1000 training hours. Each athlete sustained a median of 5.00 [5.00; 17.00] injuries or 0.96 injuries per 1000 training hours (0.27; 2.70). An amount of 22.20% (*n* = 14/63, *n* = 2 missing value) remained uninjured until now. The 1.430 injuries led to a total of 786 weeks (median 9.50 [3.00; 20.00] weeks) of downtime in their main sport due to injuries and a total of 144 weeks of work/school incapacities (median 0.75 [0.50; 3.00] weeks). The longest median periods of downtime resulted from injuries to the knee (median 12.00 [6.00; 28.00] weeks) and ankle/foot (median 6.00 [4.50; 14.25] weeks).

The number of medical consultations per year due to injuries was 2.00 [1.00; 3.00], resulting in 2.93 medical consultations per 1000 training hours. An amount of 15.40% (*n* = 10) of the collective had already undergone surgery due to sports injuries, resulting in a total of 17 surgeries (0.04 operations per 1000 training hours). The most common site of surgery was the knee (58.80%). Surgery for anterior cruciate ligament (ACL) tears (23.50%) or meniscal lesions (23.50%) accounted for the largest proportion of all surgeries.

The 1430 sports injuries consisted of 72 head injuries, 38 spine injuries, 38 trunk injuries, 129 shoulder injuries, 63 elbow injuries, 624 hand/finger injuries, 139 hip/thigh injuries, 129 knee injuries, and 198 ankle/foot injuries. Table 1 shows the distribution of injuries by body region over the entire sports career. The highest prevalences were found for the distal extremities, with 43.00% hand/fingers injuries and 14.00% ankle/foot injuries.

**Figure 3:**
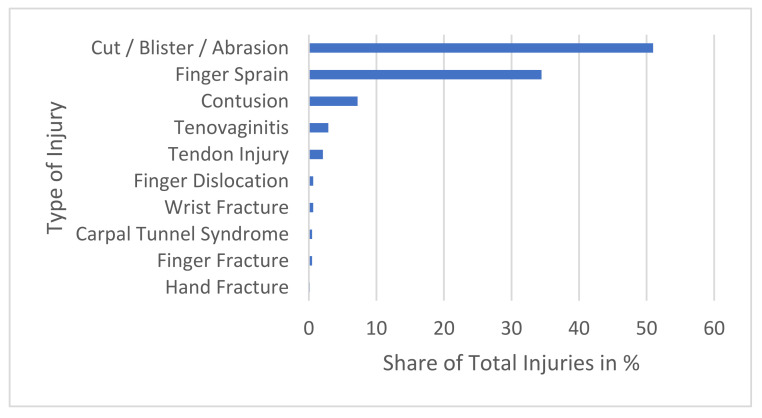
All recorded hand/fingers injuries are shown. Cut/Blister/Abrasion 51.00%, Finger Sprain 34.50%, Contusion 7.20%, Tendovaginitis 2.90%, Tendon Injury 2.10%, Finger Dislocation, Wrist Fracture 0.60% each, Carpal Tunnel Syndrome, Finger Fracture 0.50% each, Hand Fracture 0.20%.

**Figure 4:**
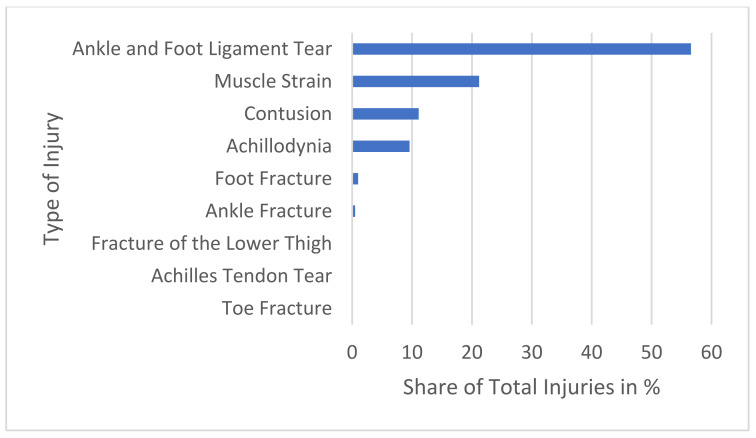
All recorded ankle/foot injuries are shown. Ankle and Foot Ligament Tear 56.60%, Muscle Strain 21.20%, Contusion 11.10%, Achillodynia 9.60%, Foot Fracture 1.00%, Ankle Fracture 0.50%, Fracture of the Lower Thigh, Achilles Tendon Tear, Toe Fracture 0.00% each.

The recorded injury types for the predominant body regions with the highest injury prevalences (hand/fingers, ankle/foot) are shown in Figure 3 and Figure 4.

The injury types for the remaining body regions (head, spine, trunk, shoulder, elbow, hip/thigh, knee) are provided as Supplementary Material (Figures S1–S7).

Within the study population, 36.70% (*n* = 18/49; *n* = 16 missing value) stated that they tend to injure themselves during training, 38.80% (*n* = 19/49; *n* = 16 missing value) estimated that most injuries occur during competition, and 24.50% (*n* = 12/49; *n* = 16 missing value) rated this as balanced.

A total of 9.80% (*n* = 4/41; *n* = 24 missing value) of the athletes reported that most injuries occurred during deaf sports, while 48,80% (*n* = 20/41; *n* = 24 missing value) stated that most injuries occurred during hearing sports. A total of 41.50% (*n* = 17/41; *n* = 24 missing value) considered this to be balanced.

A total of 20.00% (*n* = 13) of the study participants estimated that they generally suffer more injuries than hearing athletes in the same discipline, 75.40% (*n* = 49) did not. A total of 4.60% (*n* = 3) were neutral.

Across all body regions, 92.80% of the athletes perceived little or no direct correlation between their hearing loss and previous sports injuries. A total of 2.20% felt there was a positive correlation, while the remaining 5.00% of responses were neutral.

### 3.2. Contact Versus Non-Contact Sports

Within the study population, *n* = 28 (43.10%) athletes participated in contact or collision sports (soccer, handball, basketball, judo, karate), while *n* = 37 (56.90%) were affiliated to non-contact sports (athletics, beach volleyball, motor sports, tennis, sport shooting, swimming, triathlon, alpine skiing, table tennis, badminton, marathon, bowling, golf).

**Figure 5:**
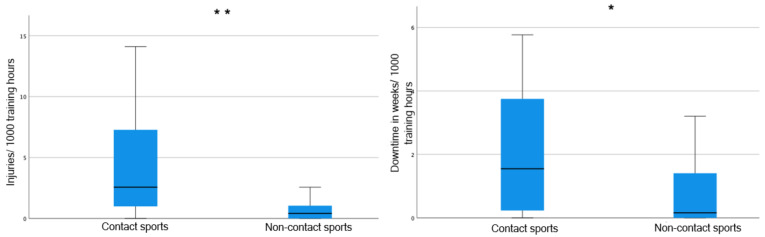
Contact sports versus non-contact sports regarding the total injuries (**left**) with highly significant difference (**, *p* < 0.001) and downtime for the main sports (**right**) with significant difference (*, *p* = 0.011), in each case/1000 training hours.

Deaf contact sport athletes had significantly more injuries per 1000 training hours in all body regions and longer downtimes per 1000 training hours than non-contact sport athletes (5.66 versus 1.28 injuries/1000 training hours; *p* < 0.001, r = 0.49; 2.45 versus 1.11 weeks/1000 training hours; *p* = 0.011, r = 0.32; Figure 5).

**Figure 6:**
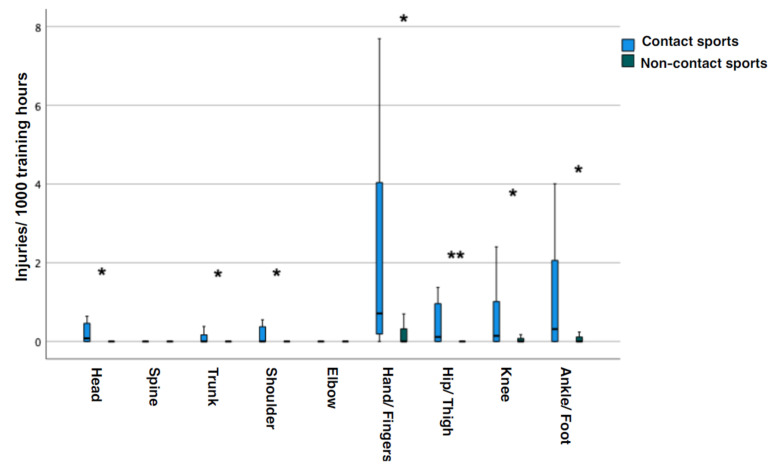
Contact sports versus non-contact sports for injuries by body region per 1000 training hours. Boxplots contain median and first/third quartile; boundaries of the whiskers are minimum/maximum values of the data set. Significant group differences (*) for head (*p* = 0.002), trunk (*p* = 0.010), shoulder (*p* = 0.024), hand/fingers (*p* = 0.001), knee (*p* = 0.010), ankle/foot (*p* = 0.002). Highly significant difference (**) for hip/thigh (*p* < 0.001). No significance for spine and elbow (*p* > 0.050 each).

This difference was confirmed by analysis of the respective injuries in almost all separate body regions except spine and elbow (head *p* = 0.002; trunk *p* = 0.010; shoulder *p* = 0.024; hand/fingers *p* = 0.001; hip/thigh *p* < 0.001; knee *p* = 0.010; ankle/foot *p* = 0.002; spine and elbow *p* > 0.05 each; Figure 6).

The frequency of incapacities for work/school was also significantly higher for deaf contact athletes than for non-contact athletes (0.29 versus 0.12 incapacities for work/school per 1000 training hours; *p* = 0.005, r = 0.35). No group difference was found for medical consultations in an average year (*p* > 0.050).

### 3.3. Supervision by an OSP

A total of 35.50% (*n* = 22/62; *n* = 3 missing value) subjects were additionally supported regionally by a licensed OSP.

This subgroup suffered significantly fewer total injuries per 1000 training hours than athletes without this support (0.92 versus 4.38 injuries/1000 training hours, *p* = 0.004, r = 0.37).

However, no group differences were found regarding downtime for the main sports, incapacity for work/school, and medical consultations (*p* > 0.050 each).

### 3.4. Assistive Hearing Devices

A total of 25.60% (*n* = 11/43, *n* = 22 missing value) of the subjects reported to use assistive hearing devices in deaf sports. Since assistive hearing devices are not allowed in deaf sports competitions according to the ICSD guidelines [11], these data refer only to training activities.

In hearing sports, however, 74.40% (*n* = 29/39, *n* = 26 missing value) of athletes use assistive devices during training and 59.00% (*n* = 23/39, *n* = 26 missing value) also use them during hearing competitions.

Regarding training activity in deaf sports, the total number of injuries was higher for athletes not using assistive devices, with 3.93 injuries/1000 training hours compared to 1.10 injuries/1000 training hours for athletes using assistive devices. However, this result was not significant (*p* = 0.087). For the major sports, there was significantly longer downtime in the group of athletes without assistive devices, with 2.29 weeks/1000 h of training compared to 0.96 weeks/1000 h of training for athletes with assistive devices (*p* = 0.045, r = 0.31). No significant differences were found for incapacities for work/school and medical consultations (*p* > 0.050 each).

## 4. Discussion

The main objective of this study was to collect epidemiological data on injuries in a representative population of deaf squad athletes. This research aims to analyze the influence of the variables contact sports, supervision by an OSP, and use of hearing aids on injury rates, downtimes, incapacities for work, and medical consultations. As the most important findings, a large proportion of deaf squad athletes are subject to a double exposure with training sessions in both deaf and hearing sports, and significantly more competitions in hearing sports. A relevant number of injuries were found in all body regions, resulting in significant downtimes, work incapacities, and medical consultations. Contact sports athletes are more frequently injured and have longer downtimes as well as more work incapacities. Athletes supervised by an OSP were less likely to be injured, and athletes not using assistive hearing devices during training in deaf sports had a higher total number of injuries and longer downtimes. Statistical analysis of effect sizes by Pearson correlation showed a medium effect according to Cohen et al. [52] for all significant results. Diverging results were found in the athletes’ subjective assessment of a correlation between their hearing loss and sustention of injuries.

In 2006, Palmer et al. characterized the deaf athlete in terms of communication, governing deaf sports organizations, and medical issues [1]. Deaf athletes may have a specific diagnosis for the cause of their hearing loss, such as congenital syndromes, infections during pregnancy, or sudden hearing loss without recovery, that must be taken into consideration by the medical staff. The authors state that there is almost no literature on medical considerations for the athlete with a hearing loss. The present study was able to investigate a relevant number of deaf squad athletes regarding epidemiology of injuries and overuse syndromes as the first of its kind.

Meiworm et al. reported on the medical care and the epidemiology of diseases of the German National Deaflympic Team (*n* = 87 athletes in 15 different disciplines) during the 19th summer Deaflympics in Rome 2001 [54]. Within 11 days of competition, a total of 186 medical treatments were documented. Similarly to the present study, the study population was heterogenous regarding sports disciplines. Regarding injuries and overuse syndromes, lower extremity was most frequently affected with 51.80% of all injuries. In comparison, the highest injury prevalences in the present study were found for the extremities, with 43.00% of all injuries to the hand and 14.00% to the foot. Tendon injuries and tendinitis were particularly common in the German National Deaflympic Team, as well as functional complaints of the spine. These results were confirmed by the present study; ligament injuries and tendinitis were reported most within the body areas ankle/foot (56.60%) and elbow (44.40%), and in 86.80% lumbago was stated in the body area spine.

Frequencies and distribution pattern of injuries and overuse syndromes of the deaf squad athletes in the present study are comparable with the results of studies on major sports events in hearing and other disabled sports [24,54,55,56,57,58,59]. However, the present study recorded injuries and overuse syndromes over whole career periods. The results of general epidemiological studies on injury frequencies in sports are supported as well by our findings in deaf competitive sports [60,61,62]. Injuries to the upper and lower extremities occur in a typical ratio, and a certain distribution pattern in favor of the distal extremities was found. As expected, minor structural pathologies like strains, contusions, and abrasions as well as functional pathologies occurred most.

Within the subgroup of head injuries, concussion was observed in a relevant number of cases with a total of 17/72 head injuries (23.60%) and 1.20% of all injuries reported. In 2021, Brancaleone examined the concussion epidemiology in deaf athletes compared with hearing athletes [63]. *n* = 693 deaf athletes and *n* = 1284 hearing athletes having participated in collegiate athletics were included. The incidence of concussion was 4.30% in deaf athletes and 8.10% in hearing athletes, respectively. This difference was not significant. Consequently, the hearing impairment obviously did not lead to a higher incidence of concussions. On the other hand, deaf athletes were shown to have poorer knowledge of concussions during sports activities in another investigation [64], so that concussion educational interventions for deaf athletes were recommended. The concussion incidence in the present study was remarkably lower. This can be explained by a larger proportion of team contact sports (e.g., soccer) in the study population examined by Brancaleone et al.

As deaf squad athletes are subject to a double exposure in deaf and hearing sports, it was deduced that this—in combination with the hearing loss itself, communicative challenges, and balance and posture difficulties—may result in a higher incidence of injuries and particular injury patterns or localizations [1,17,31]. However, the results of the present study reveal that the deaf athlete does not seem to be subject to a generally increased risk of injury, as the overall injury frequencies do not exceed the incidences retrieved from the literature on athletes in hearing sports [60,61,62].

Contact sports were associated with higher injury rates and longer downtimes in the present study, and team contact sports like soccer (24.60%) and handball (13.80%) accounted for the largest proportion of contact sports in the study population. Additionally, athletes not using assistive hearing devices during training had a higher total number of injuries and longer downtimes. Although these correlations must be interpreted carefully, it may be hypothesized that communicative deficits of deaf athletes in team contact sports may be responsible for more uncontrolled collisions. Most likely, cognitive factors like general attention, sound (direction) detection, or facilitated communication may be an explanation for this effect. In the literature, there are inconsistent data regarding positive effects of assistive hearing devices on physical activity and sports, and future research should be undertaken [65]. Consequently, the use of assistive hearing devices during both deaf and hearing sports training should be recommended. Pearson correlation showed a medium effect size for these results, emphasizing the clinical relevance of the group differences regarding contact sports and use of hearing aids. On the other hand, it is well known that contact and team sports athletes have a similarly higher injury incidence in hearing sports compared to non-contact and individual sports [61,66]. Regarding the involvement of the single body regions in the present study it must be assumed that the dominance of certain body regions can be attributed to single sports disciplines. Common injuries in soccer affect the lower extremities, including groin, knee, and ankle/foot, for example [67]. Typical injuries in handball involve hip/thigh, knee, and ankle/foot, as well as the shoulder [68]. Due to the heterogeneity of sports disciplines and subsequent small sub-groups in the present study, the results were not further analyzed regarding differences of outcome parameters between the sports disciplines. Consequently, the results of injury rates of the single body regions must be interpreted carefully, and no interpretation of these results should be conducted.

Supervision by an OSP was associated with lower injury frequencies in the study population. OSPs are support facilities for athletes in the Olympic, para-Olympic, and Deaflympic disciplines. Their main task is to ensure high-quality complex sports medicine, physiotherapy, training and exercise science, and social, psychological as well as nutritional care. The lower injury frequencies may be explained by advanced injury knowledge due to educational offers by the OSP, injury prevention and training programs containing recovery phases and additional athletics, and balance training accompanied by OSP coaches. A medium effect size revealed by Pearson correlation for this group difference underlines its practical significance. As supervision by an OSP is generally open to all deaf squad athletes, but only 35.50% of this study population were affiliated with an OSP, efforts should be aimed at connecting a higher number of athletes to their regional OSP. A consecutively increasing professionalization of deaf sports may thereby raise the athletes’ performance levels [54]. Future investigations may answer the question of how far supervision by an OSP might influence psychological or social variables in deaf or hard of hearing athletes; corresponding literature is not available up to now.

An amount of 20.00% of athletes estimated that they generally suffer injuries more frequently than hearing athletes, whereas 92.80% perceived little or no direct correlation between their hearing loss and previous sports injuries. The results on injury frequencies compared to the available literature show that a generally increased risk of injury does not seem to exist. The diverging subjective evaluations of the athletes may be attributed to their self-awareness and belonging to the deaf culture [2]. This unique cultural identity of being deaf involves considerably more than just competition [9,69]. According to Kurkova et al. and Palmer et al., the deaf athlete is physically able-bodied and can compete without marked restrictions [1,9]. This self-awareness must also be considered when accompanying these athletes, especially as a hearing coach, physician, or physiotherapist.

### Limitations

Limitations of the present study include its retrospective design and recall bias, as the study parameters requested covered the entire athletic career of the athletes. Due to the use of an online questionnaire, the response rate was only 38.20%. Both the recall bias and the response rate are to be expected in this type of survey [70,71]. Additionally, the number of questions (time to answer all questions approximately 20 min) may have contributed to the low response rate. The survey was self-developed according to the requirements of the present study. Therefore, a scientific validation of the questionnaire used is not available. On the other hand, the design and type of questions are based on similar questionnaires used in previous studies of the study group and have proven their clinical applicability [37,38,39,40,41]. Other limitations include the lack of a control group and sample size calculation due to the limited and exclusive study population, and a heterogenous number of sports disciplines. On the other hand, for the first time, a representative study population of deaf squad athletes—as a self-contained community—and their injury epidemiology could be included and systematically evaluated.

## 5. Conclusions

This study is the first to systematically provide epidemiological data on sports injuries in elite deaf athletes. The majority are exposed to a double burden of additional training and competition in hearing sports. This must be considered when working with these athletes. Deaf competitive athletes suffer a relevant number of injuries that result in significant downtime. Contact sports athletes are more likely to be injured and have longer periods of downtime, especially in team sports, which could be attributed to hearing loss. The athletes’ awareness of higher injury rates in deaf contact sports itself may help to reduce the occurrence of injuries of the predominant regions (hands/fingers, hip/thigh), and educational and psychological means may therefore be appropriate. However, the use of assistive hearing devices during training could have a positive impact on injury rates and downtime. As the use of hearing devices in competitions is—up to now—against valid ICSD guidelines, it cannot be encouraged for competitions in deaf sports. It could be a viable option in the future, but stronger evidence underlining the positive effect of assistive hearing devices will be needed. Supervision by an OSP seems to have a protective effect, so athletes should consider taking advantage of this opportunity. In addition to the statistical significance of the results, medium effect sizes demonstrate their practical relevance. Better information policy for athletes and geographical increase in contact points to an OSP are desirable. The discrepant results regarding the subjective assessment of the role of hearing loss in injury susceptibility must be placed in the context of the self-perception of the Deaf community.

In conclusion, the results of this study may help general practitioners, sports physicians, and physiotherapists to understand deaf squad athletes as high-performing professional athletes and to improve the treatment, rehabilitation, and integration of deaf athletes into hearing sports, despite communication challenges. Future study efforts should include a scientific comparison of injury patterns in deaf or hard of hearing athletes with a hearing control group in larger study populations, to understand whether unique factors are linked to hearing loss in sports and physical activity.

## Figures and Tables

**Table 1:** All recorded injuries to the nine body regions, number of athletes with at least one injury to the respective body region, and injuries/1000 training hours.

Body Region	Total Number of Injuries (%)	Number of Athletes with Injuries (%)	Injuries/1000 Training Hours
Head	72 (5.00)	22 (34.90)	0.15
Spine	38 (2.70)	13 (22.80)	0.09
Trunk	38 (2.70)	10 (17.90)	0.09
Shoulder	129 (9.00)	21 (37.50)	0.31
Elbow	63 (4.40)	11 (19.60)	0.15
Hand/Fingers	624 (43.60)	31 (55.40)	1.48
Hip/Thigh	139 (9.70)	16 (29.10)	0.33
Knee	129 (9.00)	29 (53.70)	0.31
Ankle/Foot	198 (13.80)	25 (46.30)	0.48
Total	1430 (100.00)	178	3.07

## Data Availability

The original contributions presented in this study are included in the article/Supplementary Materials. Further inquiries can be directed to the corresponding author(s).

## Supplementary Materials

The following supporting information can be downloaded at: https://www.mdpi.com/article/10.3390/sports13020043/s1, Figure S1. All recorded head injuries are shown. Figure S2. All recorded spine injuries are shown. Figure S3. All recorded trunk injuries are shown. Figure S4. All recorded shoulder injuries are shown. Figure S5. All recorded elbow injuries are shown. Figure S6. All recorded hip/thigh injuries are shown. Figure S7. All recorded knee injuries are shown. Online-Questionnaire_German. Online-based questionnaire as raw version.
