# Supplementary material for: Sports-Related Injuries in Deaf Competitive Squad Athletes—Results of a Retrospective Self-Assessment
Source: Sports (Basel). 2025 Feb 6;13(2):43. doi: 10.3390/sports13020043 (PMC11861969; doi:10.3390/sports13020043)
Supplement: Supplementary file 1 [file sports-13-00043-s001.zip › Online-Questionnaire_German.pdf]

Online-Fragebogen Injuries In Deaf Athletes

- *Demographie* (Geschlecht, Geb-Datum/Alter, Größe, Gewicht)
- *Gehörlosensportler* ja/nein
- *Chronische Erkrankungen*, die Sie in der Ausübung Ihres Sportes beeinträchtigen welche
- *Allgemeine Infos zum Sport*
  - Sportart/Disziplin seit … Jahren
  - Kaderathlet ja/nein
  - Kaderstatus (DK, EK, SK, NK2)
  - Jemals Nachwuchselite-Förderung (NEF)erhalten ja/nein
  - Betreuung durch OSP ja/nein
  - Stattgehabte oder bevorstehende EM-, WM- und/oder Deaflympics Teilnahme
  - Wettkämpfe mit Hörenden Athleten ja/nein
  - Anzahl Vereine, bei Gehörlosen zusätzlich Hörenden-Verein ja/nein
  - Liga/Klasse der Vereine gehörslos und hörend
  - Andere Sportarten ja/nein/welche/Stunden pro Woche
  - Trägst du in deiner Sportart Schutzausrüstung? Ja/nein/welche
- *Training*
  - Aufwärmzeit (min/Training)
  - Anzahl Trainings-Einheiten/Woche
  - Anzahl Trainings-Stunden/Woche
  - Trainer: hörend/gehörlos/beides
- *Verletzungen allgemein*
  - Wie oft suchst du im Durchschnitt wegen einer Verletzung in deiner Hauptsportart pro Jahr einen Arzt auf?
  - Wie oft warst du wegen Verletzungen in deiner Hauptsportart arbeitsunfähig? Im Durchschnitt für wie viele Tage?
  - Wie oft warst du wegen einer Verletzung in deiner Hauptsportart in stationärer Behandlung?
  - Wie viele Wochen insgesamt konntest du auf Grund von Verletzungen in deiner Hauptsportart diese nicht ausüben?
  - Wie oft wurdest du auf Grund von Verletzungen in deiner Hauptsportart bereits operiert? Falls ja was wurde operiert (Verletzung/OP)?

- *Verletzungen konkret*

Kopf:

Welche und wie viele Kopfverletzungen traten bei Dir auf? (Zahl nennen)

Schürfwunde:	□ Niemals	□ Ja, ___ mal
Platzwunde:	□ Niemals	□ Ja, ___ mal
Gehirnerschütterung:	□ Niemals	□ Ja, ___ mal
Hirnblutung:	□ Niemals	□ Ja, ___ mal
Nase gebrochen:	□ Niemals	□ Ja, ___ mal
Schädelbruch:	□ Niemals	□ Ja, ___ mal
Zungenbiss:	□ Niemals	□ Ja, ___ mal

Lippenbiss: □ Niemals □ Ja, ___ mal
Nasenbluten: □ Niemals □ Ja, ___ mal
Schnitte: □ Niemals □ Ja, ___ mal
Andere Verletzungen: ____

Wie oft warst Du wegen Kopfverletzungen in ärztlicher Behandlung?

□ Niemals □ Ja, ___ mal

Wie viele Wochen hast Du wegen Kopfverletzungen dein Training pausiert?

___ Wochen

In wie vielen Prozent der Fälle führst du die genannten Verletzungen auf deine Hörbeeinträchtigung zurück? __ Prozent

Wirbelsäule:

Wie oft hast Du durchschnittlich Rückenschmerzen?

nie ◆————————◆ immer

Wie stark sind die Schmerzen?

Keine Schmerzen ◆————————◆ Stärkste vorstellbare Schmerzen

Hattest Du schon mal einen Hexenschuss (Lumbago)?

□ Niemals □ Ja, ___ mal

Hattest Du schon mal eine Wirbelblockade?

□ Niemals □ Ja, Halswirbelsäule, □ Ja, Brustwirbelsäule, □ Ja, Lendenwirbelsäule

Hattest Du schon mal einen durch einen Arzt diagnostizierten Bandscheibenvorfall?

□ Niemals □ Ja, ___ mal Halswirbelsäule, □ Ja, ___ mal Brustwirbelsäule, □ Ja, ___ mal Lendenwirbelsäule

Hattest Du schon mal einen Wirbelkörperbruch?

□ Niemals □ Ja, ___ mal Halswirbelsäule, □ Ja, ___ mal Brustwirbelsäule, □ Ja, ___ mal Lendenwirbelsäule

Wie oft warst Du wegen Wirbelsäulenverletzungen in ärztlicher Behandlung?

□ Niemals □ Ja, ___ mal

Wie viele Wochen hast Du wegen Wirbelsäulenverletzungen dein Training pausiert?

___ Wochen

In wie vielen Prozent der Fälle führst du die genannten Verletzungen auf deine Hörbeeinträchtigung zurück? __ Prozent

Rumpf:

Wie oft hast Du durchschnittlich Schmerzen im Bauch- und Rippenbereich?

nie ◆————————◆ immer

Wie stark sind die Schmerzen?

Keine Schmerzen ◆————————◆ Stärkste vorstellbare Schmerzen

Hattest Du schon mal eine Bauchmuskelzerrung?

□ Niemals □ Ja, ___ mal

Hattest Du schon mal eine Rippenprellung / -bruch?

□ Niemals □ Ja, ___ mal

Hattest Du schon mal eine Rückenprellung?

□ Niemals □ Ja, ___ mal

Wie oft warst Du wegen Kopfverletzungen in ärztlicher Behandlung?

□ Niemals □ Ja, ___ mal

Wie viele Wochen hast Du wegen Kopfverletzungen dein Training pausiert?

___ Wochen ___

In wie vielen Prozent der Fälle führst du die genannten Verletzungen auf deine Hörbeeinträchtigung zurück?

__ Prozent

Schulter

Wie oft hast Du durchschnittlich Schulterschmerzen?

nie ◆————————◆ immer

Wie stark sind die Schmerzen?

Keine Schmerzen ◆————————◆ Stärkste vorstellbare Schmerzen

Welche und wie viele Schulterverletzungen traten bei Dir auf? (genaue Zahl nennen)

Prellung	□ Niemals □ Ja, __ mal
Muskelzerrung	□ Niemals □ Ja, __ mal
Schulterluxation (Auskugeln)	□ Niemals □ Ja, __ mal
Schultersteife („Frozen shoulder")	□ Niemals □ Ja, __ mal
Bizepssehnenabriss	□ Niemals □ Ja, __ mal
Engpass-(Impingement-) Syndrom	□ Niemals □ Ja, __ mal
Rotatorenmanschettenruptur	□ Niemals □ Ja, __ mal

Wie oft warst Du wegen Schulterverletzungen in ärztlicher Behandlung?

□ Niemals □ Ja, ___ mal

Wie viele Wochen hast Du wegen Schulterverletzungen dein Training pausiert?

___ Wochen ___

In wie vielen Prozent der Fälle führst du die genannten Verletzungen auf deine Hörbeeinträchtigung zurück?

__ Prozent

Ellenbogengelenk:

Wie oft hast Du durchschnittlich Ellenbogenschmerzen?

nie ◆————————◆ immer

Wie stark sind die Schmerzen?

Keine Schmerzen ◆————————◆ Stärkste vorstellbare Schmerzen

Welche und wie viele Ellenbogenverletzungen traten bei Dir auf? (genaue Zahl nennen)

Ellenbogenprellung	□ Niemals □ Ja, ___ mal

Ellenbogenfrakturbruch ◻ Niemals ◻ Ja, ___ mal
Ellenbogenluxation (Ausrenkung) ◻ Niemals ◻ Ja, ___ mal
Tennisellenbogen ◻ Niemals ◻ Ja, ___ mal
Golferellenbogen ◻ Niemals ◻ Ja, ___ mal
Sehnenscheidenentzündung ◻ Niemals ◻ Ja, ___ mal

Wie oft warst Du wegen Ellenbogenverletzungen in ärztlicher Behandlung?

◻ Niemals ◻ Ja, ___ mal

Wie viele Wochen hast Du wegen Ellenbogenverletzungen dein Training pausiert?

___ Wochen ___

In wie vielen Prozent der Fälle führst du die genannten Verletzungen auf deine Hörbeeinträchtigung zurück?

__ Prozent

Handgelenk und Hand:

Wie oft hast Du durchschnittlich Handgelenksschmerzen?

nie ◆———————◆ immer

Wie stark sind die Schmerzen?

Keine Schmerzen ◆———————◆ Stärkste vorstellbare Schmerzen

Welche und wie viele Finger-/Handgelenksverletzungen traten bei Dir auf? (genaue Zahl nennen)

Schnitte: ◻ Niemals ◻ Ja, ___ mal
Blasen: ◻ Niemals ◻ Ja, ___ mal
Abschürfungen: ◻ Niemals ◻ Ja, ___ mal
Handgelenksprellung: ◻ Niemals ◻ Ja, ___ mal
Handgelenksbruch: ◻ Niemals ◻ Ja, ___ mal
Handknochenbruch: ◻ Niemals ◻ Ja, ___ mal
Handsehnenverletzung: ◻ Niemals ◻ Ja, ___ mal
Sehnenscheidenentzündung: ◻ Niemals ◻ Ja, ___ mal
Fingerstauchung/Überdehnung: ◻ Niemals ◻ Ja, ___ mal
Fingerbruch: ◻ Niemals ◻ Ja, ___ mal
Fingerluxation (Ausrenkung): ◻ Niemals ◻ Ja, ___ mal
Karpaltunnelsyndrom: ◻ Niemals ◻ Ja, ___ mal

Wie oft warst Du wegen Hand- und Handgelenksverletzungen/-beschwerden in ärztlicher Behandlung?

◻ Niemals ◻ Ja, ___ mal

Wie viele Wochen hast Du wegen Hand- und Handgelenksverletzungen/-beschwerden dein Training pausiert?

___ Wochen ___

In wie vielen Prozent der Fälle führst du die genannten Verletzungen auf deine Hörbeeinträchtigung zurück?

__ Prozent

Becken, Hüfte und Oberschenkel:

Wie oft hast Du durchschnittlich Schmerzen im Becken-, Hüft- oder Oberschenkelbereich?

nie ◆———————◆ immer

Wie stark sind die Schmerzen?

Keine Schmerzen ◆———————◆ Stärkste vorstellbare Schmerzen

Welche und wie viele Becken-, Hüft- und Oberschenkelverletzungen traten bei Dir auf?

(genaue Zahl nennen)

Beckenprellung:	◻ Niemals ◻ Ja, ___ mal
Beckenbruch:	◻ Niemals ◻ Ja, ___ mal
Hüft-Impingement („Anschlagen“ an Pfanne):	◻ Niemals ◻ Ja, ___ mal
Labrum-(Gelenklippen-) Schaden:	◻ Niemals ◻ Ja, ___ mal
Oberschenkelbruch:	◻ Niemals ◻ Ja, ___ mal
Oberschenkelprellung:	◻ Niemals ◻ Ja, ___ mal
Muskelfaserriss Oberschenkel:	◻ Niemals ◻ Ja, ___ mal
Muskelzerrung Oberschenkel:	◻ Niemals ◻ Ja, ___ mal

Wie oft warst Du wegen Becken-, Hüft- und Oberschenkelverletzungen in ärztlicher Behandlung?

◻ Niemals ◻ Ja, ___ mal

Wie viele Wochen hast Du wegen Becken-, Hüft- und Oberschenkelverletzungen dein Training pausiert?

___ Wochen ___

In wie vielen Prozent der Fälle führst du die genannten Verletzungen auf deine Hörbeeinträchtigung zurück?

__ Prozent

Kniegelenk:

Wie oft hast Du durchschnittlich Knieschmerzen?

nie ◆———————◆ immer

Wie stark sind die Schmerzen?

Keine Schmerzen ◆———————◆ Stärkste vorstellbare Schmerzen

Welche und wie viele Knieverletzungen traten bei Dir auf? (genaue Zahl nennen)

Knieprellung:	◻ Niemals ◻ Ja, ___ mal
Kniedistorsion (Verdrehung):	◻ Niemals ◻ Ja, ___ mal
Kreuzbandriss:	◻ Niemals ◻ Ja, ___ mal
Seitenbandverletzungen:	◻ Niemals ◻ Ja, ___ mal
Meniskusverletzung:	◻ Niemals ◻ Ja, ___ mal
Knorpelschaden:	◻ Niemals ◻ Ja, ___ mal
Kniescheibenluxation (Herausspringen):	◻ Niemals ◻ Ja, ___ mal
Patellaspitzensyndrom:	◻ Niemals ◻ Ja, ___ mal
Sonstige Sehnenreizungen:	◻ Niemals ◻ Ja, ___ mal

Wie oft warst Du wegen Knieverletzungen in ärztlicher Behandlung?

◻ Niemals ◻ Ja, ___ mal

Wie viele Wochen hast Du wegen Knieverletzungen dein Training pausiert?

___ Wochen ___

In wie vielen Prozent der Fälle führst du die genannten Verletzungen auf deine Hörbeeinträchtigung zurück? __ Prozent

Unterschenkel, Sprunggelenk und Fuß:

Wie oft hast Du durchschnittlich Schmerzen im Unterschenkel, Fuß oder Sprunggelenk?

nie ◆————————◆ immer

Wie stark sind die Schmerzen?

Keine Schmerzen ◆————————◆ Stärkste vorstellbare Schmerzen

<u>Welche und wie viele Unterschenkel-, Sprunggelenks- und Fußverletzungen traten bei Dir auf? (genaue Zahl nennen)</u>

Unterschenkelprellung:	◻ Niemals	◻ Ja, ___ mal
Unterschenkelbruch:	◻ Niemals	◻ Ja, ___ mal
Muskelfaserriss Unterschenkel:	◻ Niemals	◻ Ja, ___ mal
Muskelfaserriss Unterschenkel:	◻ Niemals	◻ Ja, ___ mal
Achillessehnenriss:	◻ Niemals	◻ Ja, ___ mal
Achillodynie (chronische Reizung):	◻ Niemals	◻ Ja, ___ mal
Sprunggelenksbruch:	◻ Niemals	◻ Ja, ___ mal
Bänderdehnung Sprunggelenk:	◻ Niemals	◻ Ja, ___ mal
Bänderriss Sprunggelenk:	◻ Niemals	◻ Ja, ___ mal
Fraktur des Fußes:	◻ Niemals	◻ Ja, ___ mal
Fraktur der Zehen:	◻ Niemals	◻ Ja, ___ mal

Wie oft warst Du wegen Unterschenkel-, Sprunggelenks- und Fußverletzungen in ärztlicher Behandlung?

◻ Niemals ◻ Ja, ___ mal

Wie viele Wochen hast Du wegen Unterschenkel-, Sprunggelenks- und Fußverletzungen dein Training pausiert?

___ Wochen ___

In wie vielen Prozent der Fälle führst du die genannten Verletzungen auf deine Hörbeeinträchtigung zurück? __ Prozent

Gehörlosigkeit

Allgemeines

Beginn und Ursache der Hörstörung
Gehörlosigkeit angeboren/erworben
Seit wann

Grad der Hörminderung: „gemindert oder komplett taub“
Leichtgradig re/li/bds
Mittelgradig re/li/bds
Hochgradig re/li/bds
An Taubheit grenzend re/li/bds
Taub re/li/bds
Wenn beide Seiten betroffen sind, welche ist mehr betroffen
re/li

Haben Sie Ohrgeräusche
Ja/nein
Seite: re/ li
Auswahl Brummen/pfeifen/klopfen/rauschen/andere

Schwindel
Ja/nein
Auswahl Immer/gelegentlich/Sehr selten
Auswahl Schwankend/Drehend/Gangunsicherheit

Operationen bisher an Ihren Ohren
Ja/nein

Wenn ja, vor wie vielen Jahren und welche OP
Re…

Li…

Kommunikationsprofil

Welche Kommunikationsformen beherrschen Sie
Gebärdensprache
Labiolexie (Lippenlesen)
Gesprochene Sprache
„Totale Kommunikation“

Was ist Ihre bevorzugte Kommunikation im Alltag
Was ist Ihre bevorzugte Kommunikation bei Ihrer Sportart
    Kommunikation mit dem Trainer
    Kommunikation mit anderen Sportlern im Training und Wettkampf

Können Sie flüstern

Ja/nein
Können Sie laut rufen
Ja/nein

Können Sie im ruhigen Umfeld mit 2 oder mehr Personen kommunizieren (mit oder ohne Lippenlesen)
Ja/nein

Hörversorgung
Tragen Sie ein Hörgerät / Mittelohrimplantat (TORP, PORP, Soundbridge) / CI oder sind Sie anderweitig hörversorgt?
Ja/nein

Wenn ja
Hilfsmittel li
Auswahl
Hilfsmittel re
Auswahl

Wenn nein
nicht gewünscht / ist nicht möglich, weil…

wenn Sie nicht hörversorgt sind, können Sie bei Punkt XXX fortfahren (Punkt XXX überspringen)

Ist die Hörversorgung hilfreich bei gesprochener Kommunikation allgemein?
Ja/nein

Ist die Hörversorgung hilfreich für die Kommunikation beim Sport?
Ja/nein

Ist die Hörversorgung hilfreich für Wahrnehmung von Umgebungsgeräuschen beim Sport?
Ja/nein

Wie viele Stunden pro Tag tragen Sie die Hörversorgung im Alltag?
… Std/Tag

Fehlt Ihnen etwas ohne HG/CI?
Ja/nein

Tragen Sie Ihre Hörversorgung beim Training?
Ja/nein
Tragen Sie Ihre Hörversorgung im Wettkampf?
Ja /nein

Wahrnehmung

Können Sie problemlos sagen, aus welcher Richtung ein Geräusch (z.B. Signal des Trainers) kommt?
Mit Hörversorgung
Sicher/unsicher/unmöglich/keine Hörversorgung
Ohne Hörversorgung
Sicher/unsicher/unmöglich

Können Sie verschiedene Leute (z.B. Trainer oder Sportkollegen), die Sie kennen, nur anhand ihrer Stimme erkennen?
Mit Hörversorgung
Sicher/unsicher/unmöglich/keine Hörversorgung
Ohne Versorgung
Sicher/unsicher/unmöglich

Ist Ihre Hörminderung/Ertaubung im Umgang mit anderen Sportlern ein Hindernis?
Nie Manchmal regelmäßig meistens immer
Ist Ihre Hörminderung /Ertaubung im Umgang mit dem Trainer ein Hindernis?
Nie Manchmal regelmäßig meistens immer

Empfinden Sie Ihre Hörversorgung beim Sport als vorteilhaft?
Nie Manchmal regelmäßig meistens immer
