# Supplementary figures and images for: Sports-Related Injuries in Deaf Competitive Squad Athletes—Results of a Retrospective Self-Assessment
Source: Sports (Basel). 2025 Feb 6;13(2):43. doi: 10.3390/sports13020043 (PMC11861969; doi:10.3390/sports13020043)

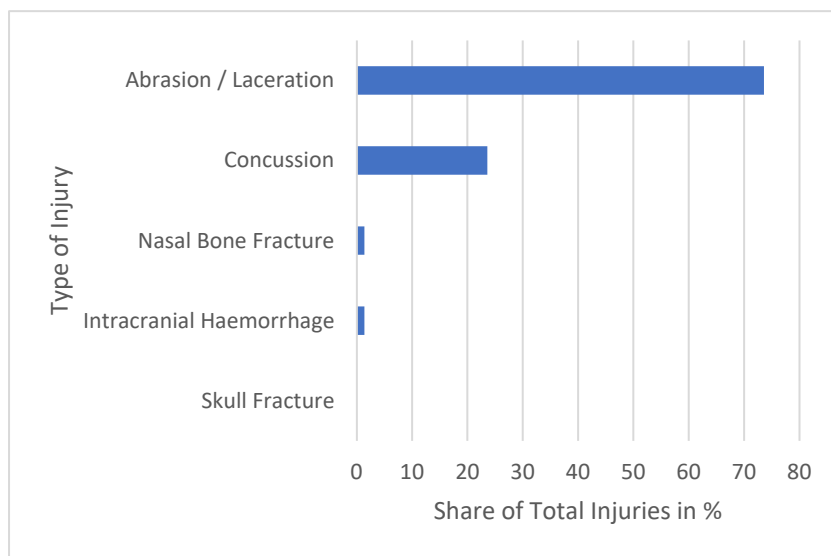

Supplement: Supplementary file 1 [file sports-13-00043-s001.zip › Figure S1_revision21.1..pdf]

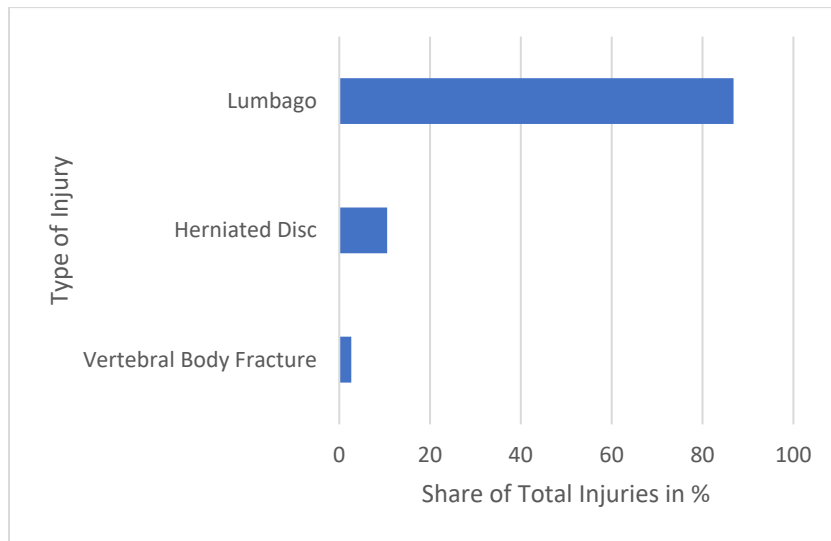

Supplement: Supplementary file 1 [file sports-13-00043-s001.zip › Figure S2_revision21.1..pdf]

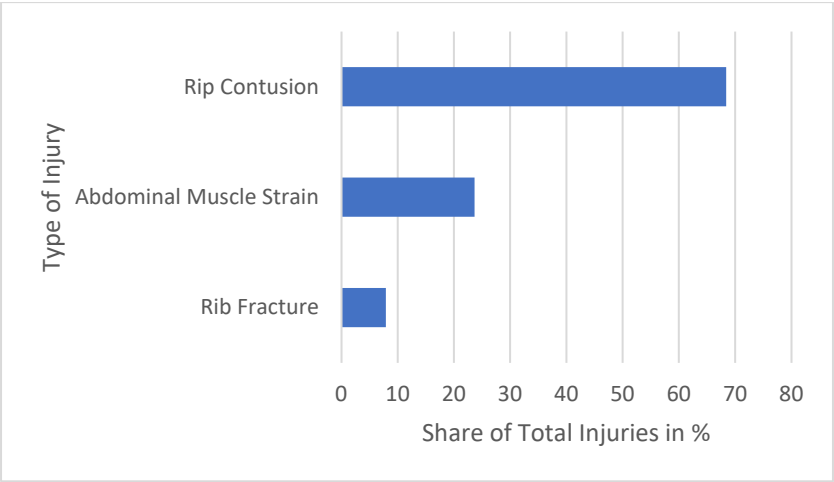

Supplement: Supplementary file 1 [file sports-13-00043-s001.zip › Figure S3_revision21.1..pdf]

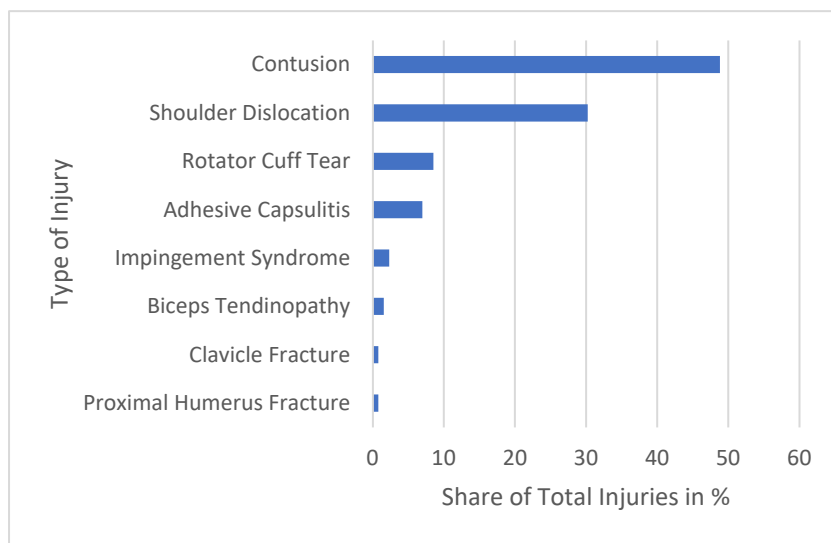

Supplement: Supplementary file 1 [file sports-13-00043-s001.zip › Figure S4_revision21.1..pdf]

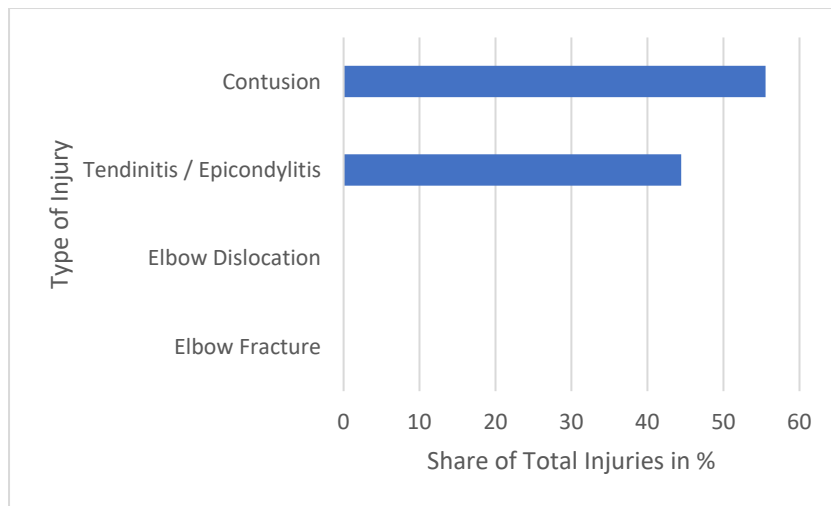

Supplement: Supplementary file 1 [file sports-13-00043-s001.zip › Figure S5_revision21.1..pdf]

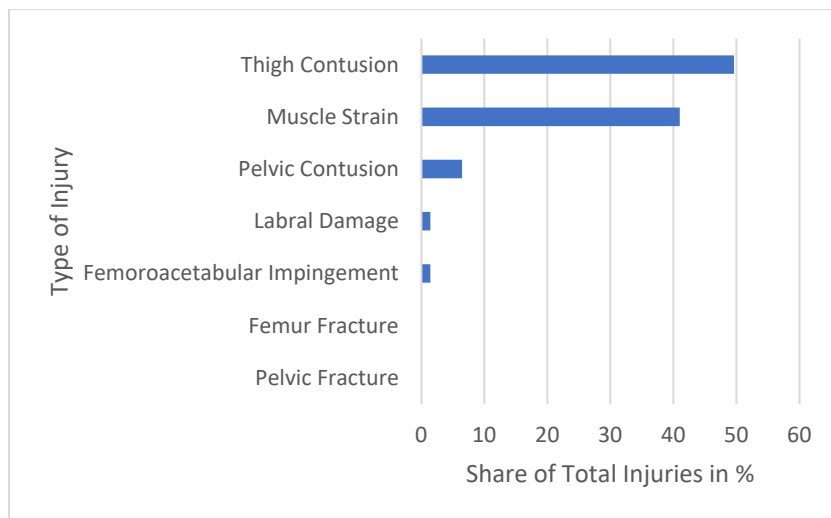

Supplement: Supplementary file 1 [file sports-13-00043-s001.zip › Figure S6_revision21.1..pdf]

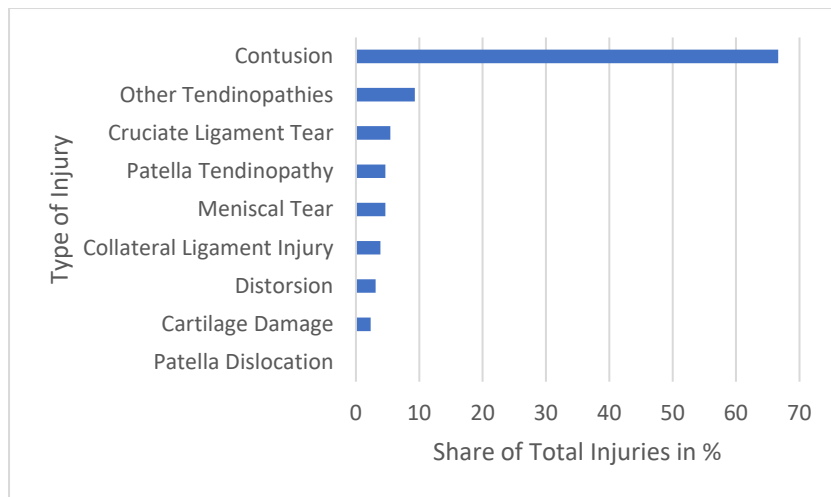

Supplement: Supplementary file 1 [file sports-13-00043-s001.zip › Figure S7_revision21.1..pdf]
